# PREFACE

**DOI:** 10.2174/157015912799362797

**Published:** 2012-03

**Authors:** Thomas E. Salt

This Editorial marks the beginning of the tenth year of publication of ***Current Neuropharmacology**.* As Chief Editor I have watched the growth and success of the journal with great pleasure. The impact of *Current Neuropharmacology* has steadily increased and the journal is freely available on PubMed Central. The standard and range of reviews and special issues consistently attains high standards. Naturally, none of this would have been possible without the dedicated efforts of authors, Editorial Board members, Special Guest Editors, anonymous reviewers, and of course the tireless efforts of Bentham Science Publishers. To all of these I express my sincere thanks.

The diversity and high standard of work published in Current Neuropharmacology is reflected in the content of the present issue of the journal. The subject matter ranges from reviews of the pharmacology of specific types of receptors (*e.g.* Therapeutic Potential of Metabotropic Glutamate Receptor Modulators by Hovelsø *et.al.*), evaluation of disease mechanisms and pharmacology (*e.g.* Effects of Cocaine on Maternal Behavior and Neurochemistry by Nephew & Febo) to reviews of clinical data (*e.g.* Clozapine-Induced Obsessive-Compulsive Symptoms in Schizophrenia: A Critical Review by Schirmbeck & Zink).

For *Current Neuropharmacology* to continue to thrive it is important that diversity and high standards are maintained. With its increasing impact and visibility, I believe that the journal offers an excellent vehicle for the publication of novel reviews and hypotheses, and I look forward to the submission and publication of novel and leading papers in the future.

